# Efficacy of the Direct Aspiration-Irrigation Maneuver for the Treatment of Chronic Subdural Hematoma: A Single Hospital's Experience

**DOI:** 10.7759/cureus.16231

**Published:** 2021-07-07

**Authors:** Zhenjiang Pan, Jing Bao, Shepeng Wei

**Affiliations:** 1 Neurosurgery, Shidong Hospital of Yangpu District, Shanghai, CHN

**Keywords:** chronic subdural hematoma, csdh, sbaid, sbid, recurrence rate

## Abstract

Objective

The traditional methods for managing symptomatic chronic subdural hematoma (CSDH) at our hospital include evacuation via single burr-hole irrigation with continuous closed subdural drainage (SBID). The single burr-hole aspiration and irrigation technique with continuous closed subdural drainage (SBAID) is an attractive alternative method. The goal of this study was to evaluate the radiographic and clinical outcomes of SBAID compared with traditional SBID methods.

Methods

A database of 51 CSDH patients treated with the SBAID method and 35 CSDH patients treated with the SBID method was compiled, and a retrospective chart review was performed. Information regarding demographics, comorbidities, presenting symptoms, and outcomes were collected. Predictors of recurrence requiring reoperation and other outcomes were analyzed.

Results

Compared to the patients in the SBID group, the patients in the SBAID group had a shorter mean duration of surgery (56.6±5.6 minutes vs 59.5±4.8 minutes, respectively, P=0.02); a shorter mean interval from procedure to discharge (6.2±1.2 days vs 6.8±1.3 days, respectively, P=0.046); no significant difference in preoperative hematoma volume (106.4±21.7 cm^3^ vs 101.3±16.3 cm^3^, respectively, P=0.25); and a smaller subdural space volume 48 hours after the operation (43.6±7.4 cm^3^ vs 47.4±9.1 cm^3^, respectively, P=0.03).

In addition, symptomatic hematoma recurrence developed in one patient in the SBAID group and five patients in the SBID group (P=0.03). The in-hospital mortality rates of the SBAID and SBID groups were 2% (1 of 51) and 6% (2 of 35), respectively; this difference was not statistically significant (P=0.35).

Conclusions

The SBAID method results in a remarkably low recurrence rate and good outcomes. This method should be considered for patients presenting with symptomatic CSDHs.

## Introduction

Chronic subdural hematoma (CSDH) is one of the most commonly encountered diseases in clinical neurosurgical practice. CSDH can be defined as a mainly hypodense or isodense crescentic collection along the cerebral convexity on head computed tomography (CT), characterized by an encapsulated accumulation of fluid, blood, and blood degradation products layered between the arachnoid and dura mater on the brain's surface [1].

As a result of the combination of an aging population and the increasing use of anticoagulant and antiplatelet medications, the incidence of CSDH is expected to increase over the course of the next 25-30 years [2-3]; the current incidence is approximately five to 13 per 100 000 per year in the general population and is higher for those aged 70 years and older (58 per 100 000 per year) [4-7].

Multiple surgical techniques have been frequently described in the literature, including closed-system drainage after a one- or two-burr hole craniostomy [8-9], twist-drill craniostomy with or without drainage, craniotomy, and evacuation of the CSDH and its surrounding membranes [10], and many additional but less commonly practiced methods [11-13]. However, there is no universal consensus regarding the optimal surgical procedure for treating CSDH [14]. Although CSDH is usually not a life-threatening condition, its clinical course is not benign [15], especially when subdural hematoma recurs after surgery.

Modified burr-hole drainage for the treatment of CSDHs was introduced in 2016 in our hospital. This method is a variation of burr-hole drainage with irrigation that adds aspiration during the irrigation process and allows large debris from the contents of the CSDH to be suctioned out. This operation is called single burr-hole aspiration and irrigation with continuous closed subdural drainage (SBAID). Our previous method is called single burr-hole irrigation with continuous closed subdural drainage (SBID).

Before November 2016, SBID was always used to treat CSDH at our hospital. Significant pneumocephalus and more frequent recurrence prompted us to use SBAID as our treatment for CSDH unless the CSDH is limited to the parietal area. SBAID can provide a complete replacement of the hematoma with normal saline with little debris left in the subdural space. Moreover, removal of the drainage catheter two days later does not cause any additional brain injuries.

This present study represents a single hospital's experience with the treatment of CSDH and includes patients who were treated and followed over a period of one year. We believe that SBAID has proven to be simple and effective, with a relatively short operative time and a low risk of intra- and postoperative complications.

The present study reviews our experience with SBAID and compares it to SBID for the treatment of CSDH. This article was previously posted to the Research Square server on March 4, 2021.

## Materials and methods

Patient selection and data

The patients in this study were inpatients at Shidong Hospital from March 2014 to December 2018 with a diagnosis of CSDH based on standard radiographic characteristics.

SBAID procedures were performed between November 2016 and December 2018. To minimize selection bias, we reviewed the SBID cases from a period when SBAID was not performed. SBID procedures were performed between March 2014 and October 2016. In other words, we changed the surgical method to SPAID after October 2016.

The Shidong Hospital of Yangpu District's Research Ethics Board approved the study. Written informed consent was obtained from all patients or their legally authorized representatives before participating in the study. All methods were performed in accordance with relevant guidelines and regulations.

The patients had a postoperative follow-up duration of five to 55 weeks. The primary endpoint was recurrence requiring reoperation within two months. The secondary endpoints were complications requiring hospital admission and mortality.

Data from a total of 86 patients who underwent 108 procedures are presented in this series. General patient data, including age, sex, whether the patient was receiving anticoagulation therapy, and other risk factors, as well as preoperative symptoms, length of stay, follow-up interval, and stability of CSDH at follow-up, were analyzed, and the findings of preoperative 64-slice spiral head CT studies were collected. Postoperative symptoms, findings of postoperative 64-slice spiral head CT scans, complications, and rates of recurrence or remnant hematoma were also captured. The Coniglobus formula was used to calculate preoperative and postoperative hematoma volumes. Preoperative and 48-hour postoperative head CT scans and follow-up CT scans at the first outpatient clinical visit were used to measure the subdural volume. The endpoint of our study was the determination of residual CSDH at the first follow-up visit.

Patients were included if they had a subdural hematoma with mixed acute or subacute components or if there were separations present within the hematoma. Patients with a hematoma confined to the frontal or parietal area were excluded. All patients underwent single-hole trepanation and placement of an intracavity closed drainage system as surgical treatment. CSDH recurrence was defined as a subsequent increase in hematoma volume in the ipsilateral subdural space with neurological deficits that was followed by another operation [16].

Operative technique

The patients were placed in a supine position with the head tilted slightly to the opposite side. Most patients underwent surgical procedures under general anesthesia, but local anesthesia was performed for patients who could tolerate the operation.

**Figure 1:**
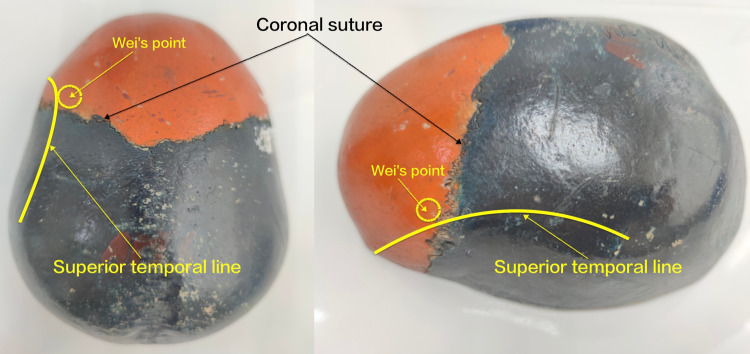
Wei's point is located 0.5 cm medial to the superior temporal line and 1 cm anterior to the coronal suture.

The SBAID procedure was performed as follows. After skin preparation, the site for trepanation was marked at Wei's point. Wei's point is located 0.5 cm medial to the superior temporal line and 1 cm anterior to the coronal suture (Figure 1).

After the dura was opened, the tip of a soft catheter (Medtronic, Minneapolis, Minnesota, USA) was then inserted in the dorsal direction to the deepest point of the hematoma cavity. We first aspirated all the liquid from the hematoma using a 50-ml syringe (5 ml per second) and then injected normal saline (room temperature) into the cavity through the burr hole. Almost simultaneously, we aspirated the content out of the cavity. Using this aspiration-irrigation technique, the subdural cavity was thoroughly washed with normal saline. The process of slow aspiration and irrigation continued until the outcoming fluid was clear. Then, the tip of the catheter was inserted into the frontal point. At this time, we began to rinse the frontal part of the cavity by injecting 200 ml of normal saline into it through the catheter. After irrigation, the tip of the catheter was inserted again into the dorsal part of the hematoma cavity. The slow irrigation-aspiration process continued until the outcoming fluid was clear again. Once the aspiration fluid was clear, the tip of the catheter was again inserted into the highest frontal point, with 3-5 cm of the catheter located in the subdural space. The rest of the catheter was then tunneled under the skin and away from the burr hole through a separate incision. Then, the patient’s head was tilted slightly to the opposite side to ensure that the burr hole was at almost the highest point. We began to inject normal saline into the subdural space through the catheter to ensure that the cavity was filled with saline instead of air before the closure of the incision. Then, the catheter was connected to a closed-system drainage bag that was placed under the patient's head for passive drainage.

In the case of bilateral CSDHs, similar steps were then followed on the other side. Drainage was usually maintained for 48 hours to collect the remaining subdural fluid with the aid of brain pulsation. During the drainage period, the patient stayed in bed until the catheter was removed.

During the SBID procedure, the patients were also positioned supine, with the head tilted obviously to the opposite side. A single 1.5-cm burr hole was drilled over the maximum width of the hematoma cavity, typically at the parietal eminence and not at Wei's point. After the dura mater was opened, the same soft catheter was placed carefully in all directions of the hematoma cavity to irrigate subdural collections with 2000-3000 ml of normal saline until the outflowing fluid was clear. Of note, at this point, we simply irrigated the subdural collections and did not aspirate the contents of the subdural space. At that time, we did not perform aspiration because our teachers at Tiantan Hospital and Huashan Hospital did not teach us that CSDH could be suctioned. The rest of the procedure was the same as that used for SBAID. The two surgical techniques were adopted by all surgeons in our department.

Postoperative head CT was routinely performed 48 hours after the operation as a baseline investigation and to exclude remnant hematoma or tension pneumocephalus. Head CT or MRI investigations were also performed at the one-month clinical follow-up.

Statistical analyses

Clinical characteristics were described using means (standard deviations) for continuous variables and numbers of patients (percentages) for categorical variables. Relationships between clinical characteristics were assessed using the Χ2 test for frequency analyses and analysis of variance for the comparison of means. The change in volume was standardized between procedures and among patients by using the percentage change in volume measured by the following equation: [1-(final volume/initial volume)] × 100.

Significance was set at a P-value of <0.05, and all tests were 2-tailed. All statistical analyses were performed using SPSS software, version 17.0.0 (SPSS Inc., Chicago, Illinois, USA).

## Results

A total of 108 operations performed on 86 patients (16 patients with bilateral CSDH and 6 patients who underwent reoperation) were included in this study. The average length of follow-up was 31.6 weeks and 29.4 weeks in the SBAID and SBAID groups, respectively.

Table 1 shows the baseline clinical characteristics of the patients with CSDH in the two groups. The two groups did not differ significantly with respect to demographic features.

**Table 1:** Demographic characteristics and presenting features of the SBAID and SBID groups SBAID: single burr-hole aspiration and irrigation technique with continuous closed subdural drainage; SBID: single burr-hole irrigation with continuous closed subdural drainage

Variable	SBAID; n=51	SBID; n=35	Difference
Average age in years (range, SD)	69 (33–96, 10.5)	65(28–85, 9.5)	P=0.33
Number of patients <35 years old (%)	1(2)	1(3)	P=0.79
Number of males (%)	32(63)	23(66)	P=0.78
History of trauma (%)	39(76)	22(63)	P=0.17
History of cerebrovascular accidents (%)	19(37)	11(31)	P=0.58
Bilateral hematoma (%)	10(20)	6(17)	P=0.77
CT image: membranous (%)	13(25)	8(23)	P=0.78
Seizure (%)	5(10)	2(6)	P=0.50
Number of patients treated with anticoagulants (%)	30(59)	16(46)	P=0.23
Neurological deficits (%)	34(67)	24(69)	P=0.85

Compared to the patients in the SBID group, the patients in the SBAID group were less likely to have a history of trauma (76% vs 63%, respectively, P=0.17); a previous history of cerebrovascular accidents (37% vs 31%, respectively, P=0.58); bilateral hematomas (20% vs 17%, respectively, P=0.77); and membranous CT images (25% vs 23%, respectively, P=0.78).

Table 2 shows the CT features of the patients with CSDH in the two groups. The two groups did not differ significantly with respect to CT features.

**Table 2:** CT features of the SBAID and SBID groups according to a modified Nakaguchi classification as described by Stanišić and Pripp Stanišić and Pripp [17] SBAID: single burr-hole aspiration and irrigation technique with continuous closed subdural drainage; SBID: single burr-hole irrigation with continuous closed subdural drainage

Type		SBAID; n=51	SBID; n=35	Difference
Homogeneous	hypodense	6 (11.8%)	4（11.4%）	P=0.96
	isodense	11 (21.6%)	8（22.9%）	P=0.89
	hyperdense	1 (2.0%)	1（2.9%）	P=0.79
Laminar (mixed)		9 (17.6%)	6（17.1%）	P=0.95
Separated subtype		12 (23.5%)	8（22.9%）	P=0.94
gradation subtype		4 (7.8%)	3（8.6%）	P=0.93
Trabecular (multilocular)		8 (15.7%)	5（14.2%）	P=0.86

Table 3 shows the clinical outcomes of the patients with CSDH in the two groups. Compared to the patients in the SBID group, the patients in the SBAID group had a shorter mean duration of surgery (56.6±5.6 minutes vs 59.5±4.8 minutes, respectively, P=0.02) and a shorter mean interval from procedure to discharge (6.2±1.2 days vs 6.8±1.3 days, respectively, P= 0.046).

**Table 3:** Clinical outcomes of patients in the SBAID and SBID groups SBAID: single burr-hole aspiration and irrigation technique with continuous closed subdural drainage; SBID: single burr-hole irrigation with continuous closed subdural drainage; CSDH: chronic subdural hematoma

Variable	SBAID; n=51	SBID; n=35	Difference
Mean duration of surgery in minutes	56.6±5.6	59.5±4.8	P=0.02
Mean interval from procedure to discharge in days	6.2±1.2	6.8±1.3	P=0.046
Average length of follow-up in weeks (range)	31.6±7.7	29.4±8.5	P=0.21
% lost to follow-up	12 (6/51)	14 (5/35)	P=0.73
% with stable CSDH at follow-up	95.5 (43/45)	90.0 (27/30)	P=0.34
Number of recurrences	1 (2%)	5 (14%)	P=0.03
Number of deaths	1 (2%)	2 (6%)	P=0.35
Number of postoperative seizures	1 (2%)	3 (9%)	P=0.15

The in-hospital mortality rates of the SBAID and SBID groups were 2% (1 of 51) and 6% (2 of 35), respectively; this difference was not statistically significant (P=0.35). The cause of death of one patient in the SBAID group, an 81-year-old man, was brain herniation caused by acute intracranial hematoma after removal of the drainage catheter. The relatives refused the request to remove the hematoma, and seven hours later, the patient died. One cause of death in the SBID group was aspiration pneumonia in a 76-year-old man. Another cause of death in the SBID group was brain herniation caused by acute subdural hematoma in an 83-year-old man for whom the family refused a further operation.

Postoperative seizures developed in one patient in the SBAID group and three patients in the SBID group (P=0.15); these patients all suffered from partial motor seizures that were managed successfully with anticonvulsant drugs (Tegretol).

All patients were reexamined postoperatively with regard to the presenting symptoms. Postoperative tension pneumocephalus was excluded in all patients in the two groups.

Table 4 shows the radiographic outcomes of the SBAID and SBID methods for the evacuation of CSDHs.

**Table 4:** Radiographic outcomes of the SBAID and SBID methods for the evacuation of CSDHs SBAID: single burr-hole aspiration and irrigation technique with continuous closed subdural drainage; SBID: single burr-hole irrigation with continuous closed subdural drainage; CSDH: chronic subdural hematoma

Radiographic variable	SBAID	SBID	P-value
Preoperative hematoma volume in cm^3^	106.4±21.7	101.3±16.3	0.25
Subdural space volume in cm^3 ^48 hours later	43.6±7.4	47.4±9.1	0.03
Air volume in cm^3 ^48 hours later	22.7±6.1	25.6±5.4	0.03
Final volume in cm^3^ 30 days later	25.6±4.5	27.4±5.7	0.13
Percentage change 30 days later (%)	74.6±7.4	71.8±8.6	0.11

Compared to the patients in the SBID group, the patients in the SBAID group had a smaller subdural space volume 48 hours after the operation (43.6±7.4 cm3 vs 47.4±9.1 cm3, respectively, P=0.03); and a significantly smaller intracranial air volume 48 hours after the operation (22.7±6.1 cm3 vs 25.6±5.4 cm3, respectively, P=0.03). 

## Discussion

Our study first presented the clinical characteristics of our patients with CSDH. Second, it introduced a direct single-burr-hole aspiration-irrigation maneuver and closed drainage strategy for the treatment of CSDHs. Third, it reported that this method achieved a remarkably low recurrence rate and good outcome.

How does SBAID reduce the recurrence rate?

The reason we achieved a much lower recurrence rate may be that less air was left in the subdural space and that complete replacement of the liquid with normal saline was achieved to the greatest extent possible.

The traditional methods for treating symptomatic CSDHs have been operative evacuation through one or two burr-hole irrigations with closed subdural drainage. Although a number of modifications of these procedures have been described, none can reduce the high recurrence rate. Reoperation has been shown to affect postoperative functional outcomes [4] and quality of life [16]. CSDH recurrence is usually defined as the reaccumulation of hematoma fluid requiring another operation [16]; defined in this way, the recurrence rates reported in the literature vary widely, from 0% to 76% [15-16]; however, the current consensus is that the reoperation rate ranges from 10% to 20% [18]. Factors that increase the risk of recurrence include drainage catheter occlusion, too much intracranial air [19], bilateral CSDHs [20-21], preoperative use of anticoagulant [22-24], and antiplatelet [22] medications, intraoperative visualization of poor brain reexpansion and thick membranes [25], postoperative persistence of midline shift [24], and the volume of the postoperative hematoma cavity [26-27], although the data for these factors come from small studies with low events-per-variable ratios and thus must be interpreted cautiously [18]. In our study, the reoperation rate in the SBID group was 14%, whereas the reoperation rate after the SBAID method was only 2% (P=0.03).

The volume of the postoperative hematoma cavity is a well-known risk factor for recurrence in patients with CSDH [26-27], and our results support this. Compared to the patients in the SBID group, the patients in the SBAID group had a smaller subdural space volume 48 hours after the operation (P=0.03). The average percentage changes after one month in the SBAID and SBID groups were 74.6% and 71.8%, respectively (p=0.11).

Both the decreased brain plasticity caused by long-term compression from hematomas and preexisting brain atrophy can affect brain reexpansion. Thus, the postoperative hematoma cavity cannot be eliminated quickly. After surgery, patients need to remain in a supine position in bed for three to seven days. With patients lying in the supine position, the effect of gravity usually causes the parietal lobe to reexpand very quickly, and the frontal space is the last part that expands. When the tip of the catheter is located in the anterior part of the cavity, the catheter does not touch the hematoma capsule during the drainage period, even when the catheter is pulled out of the cavity.

In the SBAID group, subdural collections were replaced with normal saline as much as possible via aspiration and irrigation. To effectively replace all the contents of CSDHs with normal saline, we suggest performing the aspiration-irrigation maneuver, especially aspiration, during the operation. In every patient in our series, we always found some debris in the fluid of the CSDH, most of which could only be aspirated out. Compared to the patients in the SBID group, the patients in the SBAID group had a shorter mean duration of surgery (P=0.02) and a shorter mean interval from procedure to discharge in days (P=0.046). In our opinion, the aspiration process reduces the length of the operation.

**Figure 2:**
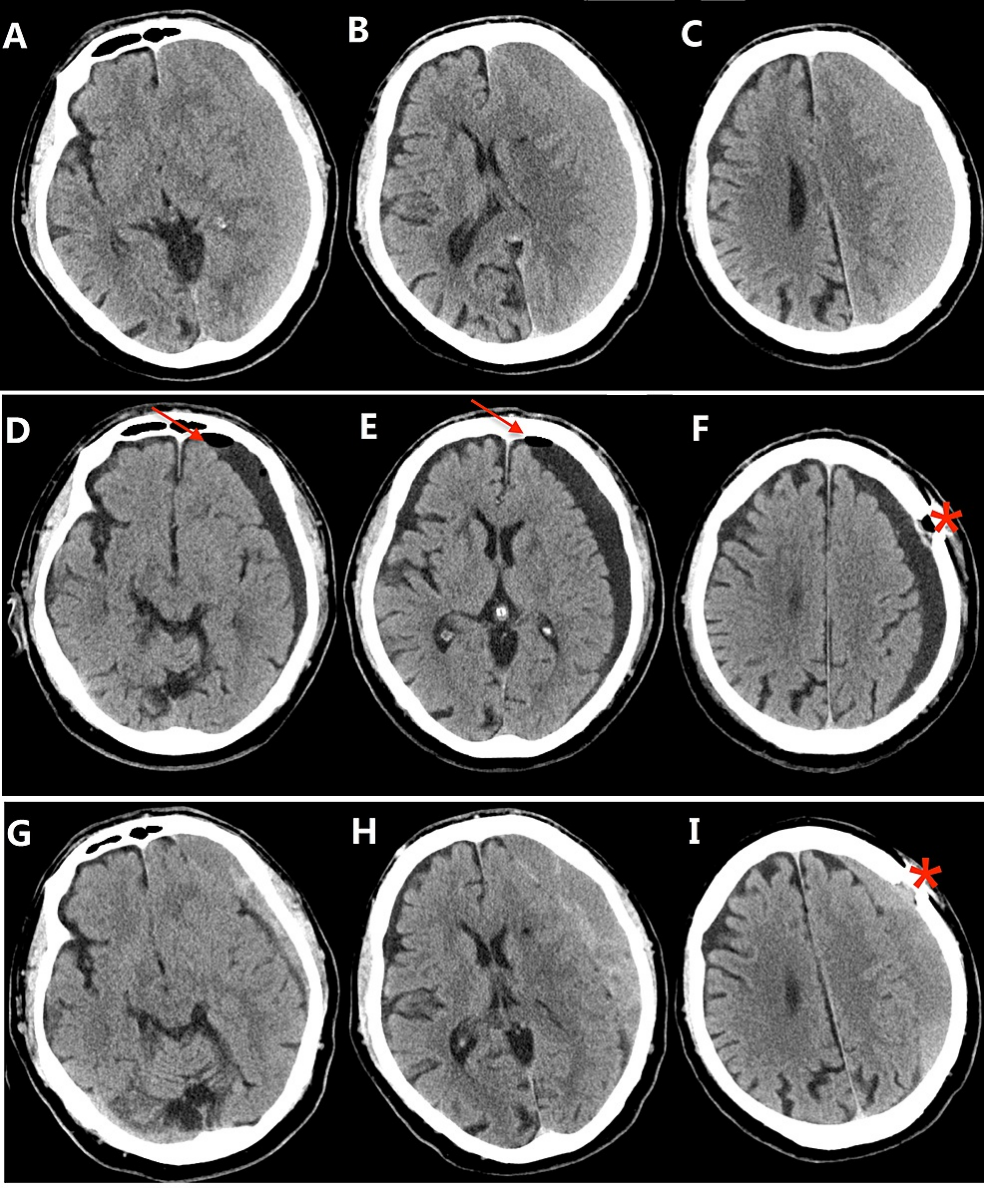
Axial CT images of a recurrent case in the SBAID group Upper row: Preoperative CT image (A, B, C) showing left-lateralized CSDH. Middle row: Postoperative CT image (D, E, F) [Day 2] showing Wei's point (＊) and indicating that the residual hematoma had shrunk and had little air (arrow) remaining in the cavity. Lower row: Postoperative CT image (G, H, I) [30 days] showing recurrent CSDH. CSDH: chronic subdural hematoma

**Figure 3:**
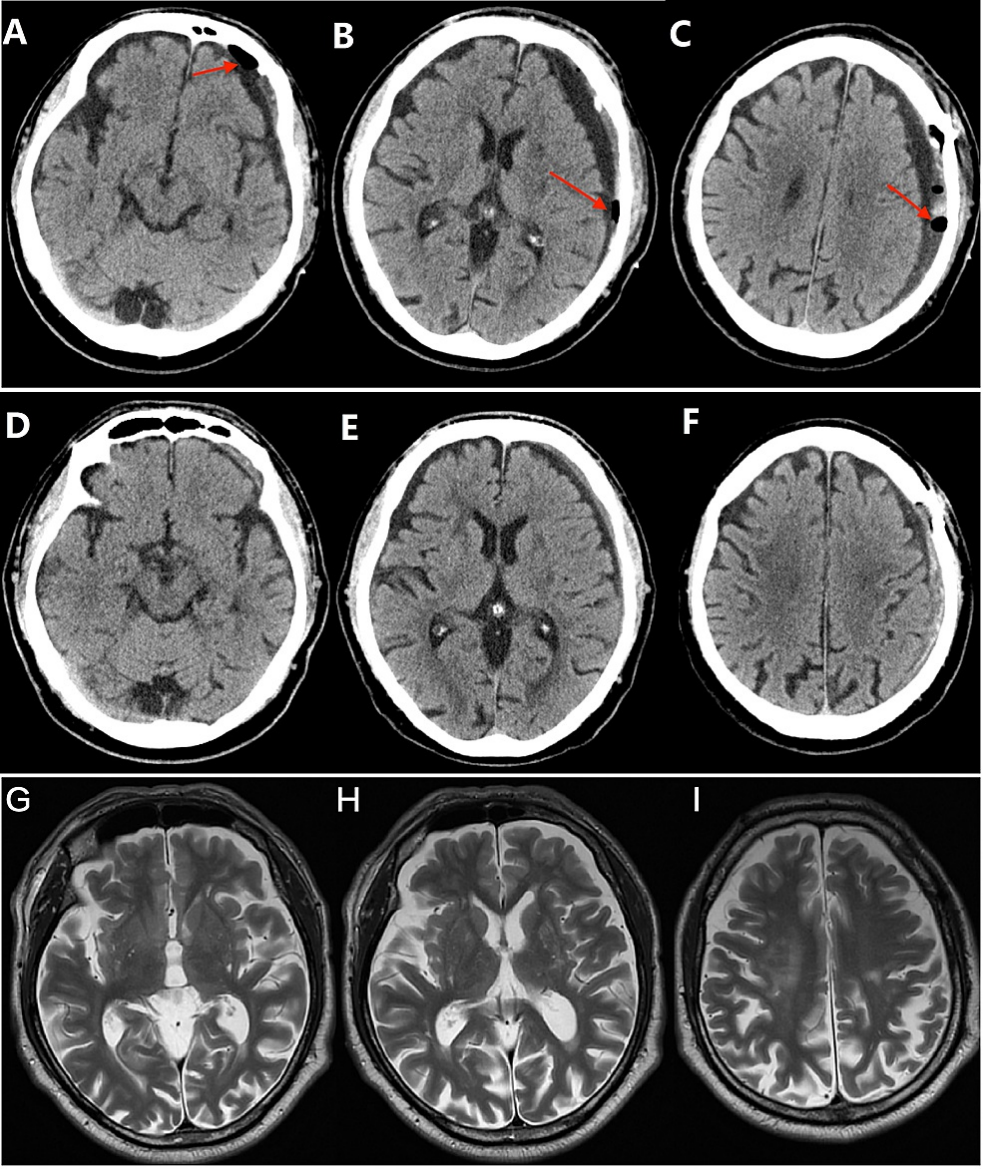
Axial CT images of the same patient in the SBAID group Upper row: Preoperative CT image (A, B, C) [Day 2] showing that the residual hematoma had shrunk and that little air was left (arrow) in the cavity. Middle row: Postoperative CT image (D, E, F) [Day 30] showing that the hematoma had almost disappeared. Lower row: Postoperative CT image (H, I, G) [1.5 years later] showing a normal brain structure. SBAID: single burr-hole aspiration and irrigation technique with continuous closed subdural drainage

The results showed that the volume of the hematoma cavity was reduced by a mean rate of 70.4%, and the recurrence rate was only 2%, which suggests that the volume of the hematoma cavity after the operation reached an optimum state through our strategy. Additionally, with our strategy, the recurrence rate in our cohort was significantly lower than that reported in the literature [4,28-30]. The reason one patient in our series experienced recurrence appears to be occlusion of the drainage catheter (Figures 2-3). The patient was cured by using a second single burr-hole aspiration and irrigation technique with continuous closed subdural drainage.

Following our strategy, aspiration is safe and effective for the replacement of CSDH contents and sharply reduces the recurrence of CSDHs. During surgery, care should be taken to aspirate the fluid of the CSDH slowly and inject normal saline into the cavity through the burr hole at the same time, ensuring that the output/input speed is equal.

In cases in which many membranes separate the hematoma into chambers in a honeycomb fashion, an open procedure cannot be avoided. However, in our series, we did not encounter any cases of multiple membranes.

The avoidance of direct contact between the catheter and the hematoma capsule may moderate the risk of postoperative seizures and limit the secondary spread of infection to intracranial compartments [31]. Four patients in our series (1 in the SBAID group and 3 in the SBID group) suffered from partial motor seizures, possibly due to injury to the inner capsule of the hematoma and the brain cortex. After three months of antiepileptic drug therapy, these patients stopped taking Tegretol and did not ever have another seizure. The placement of a closed drainage system may increase the infection rate of the hematoma cavity and wound, but only one patient in the two groups experienced fever symptoms and wound infection. The infection was cured after treatment with antibiotics and seven days of wound dressing changes. Furthermore, wound infection did not affect the outcome of CSDH.

**Figure 4:**
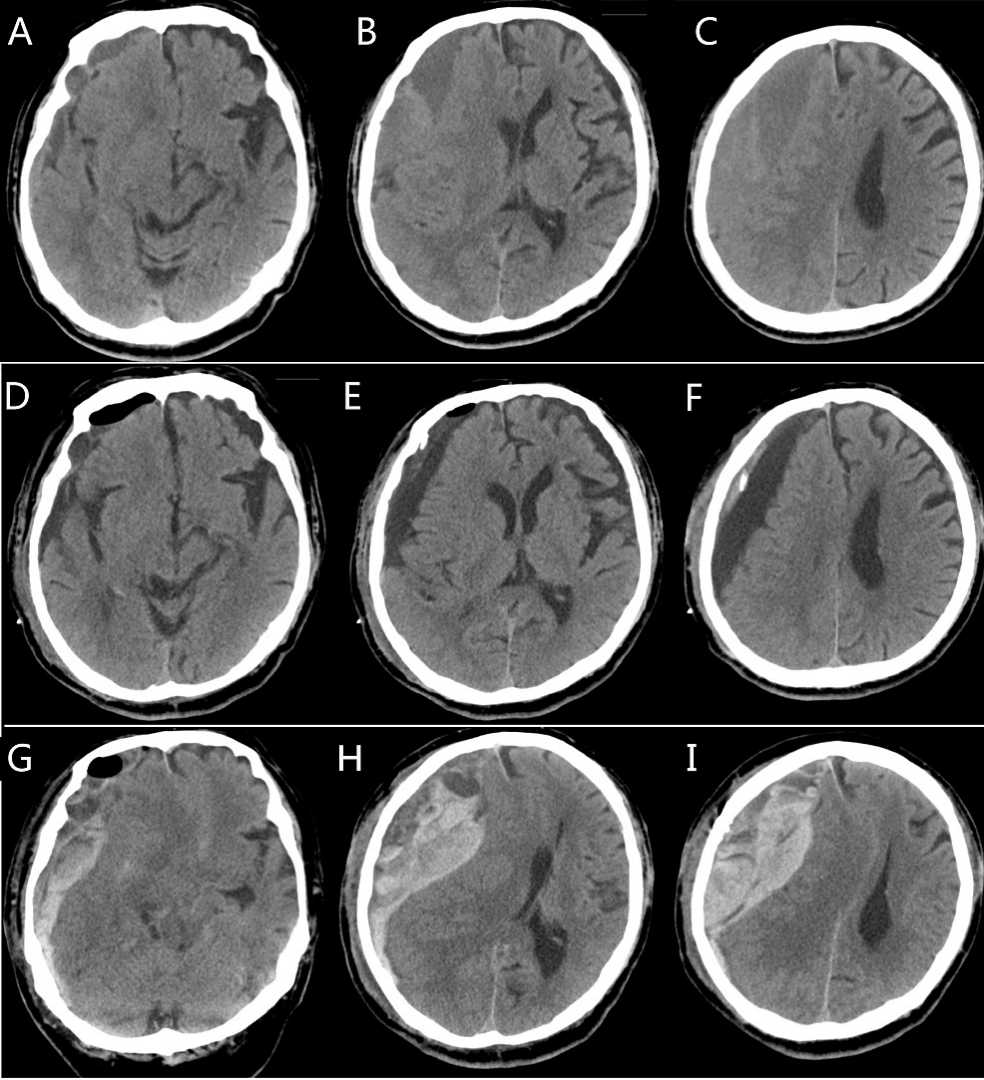
Axial CT images of a patient with complicated intracranial hematoma in the SBAID group Upper row: Preoperative CT image (A, B, C) showing right-lateralized CSDH. Middle row: Postoperative CT image (D, E, F) [day 2] showing that the residual hematoma had shrunk and that little air was left in the cavity. Lower row: Postoperative CT image (G, H, I) [4 hours after removal of the drainage catheter] showing a recurrent large acute intracranial hematoma. SBAID: single burr-hole aspiration and irrigation technique with continuous closed subdural drainage; CSDH: chronic subdural hematoma

One patient in our series suffered from an acute subdural hematoma on the second day after the operation (36 hours later). The patient presented with coma three hours after the catheter was removed. Head CT showed a large acute subdural hematoma on the same side as the CSDH. The patient's family refused to allow reoperation, and the patient died four hours later (Figure 4). The reason for this complication was not clear. The removal of the catheter may have damaged an artery of the skin, and blood may have entered the subdural space through the burr hole.

Why did we choose Wei's point?

In addition to an acute subdural hematoma, one immediate postoperative complication was tension pneumocephalus. Pneumocephalus is an independent factor for recurrence [32-34]. Some studies recommend that the patient's head should be fixed to ensure that the burr hole is always situated at the highest point so that the cavity can be filled with saline instead of air before closure [19]. We don't have references related to Wei's point. Benefiting from the inspiration of double burr holes treatment of CSDH, we tried to use the anterior hole as the only burr hole. To ensure that the burr hole was situated at the highest point, we chose Wei's point as the site of the burr hole. Just as the parietal eminence is usually the thickest part of the CSDH after the hairline, Wei's point is the thickest part of the anterior hematoma that is closest to the hairline. The burr holes of patients in the SBID group were made over the parietal eminence.

Why should we choose the aspiration method?

Certain substances (such as inflammatory mediators, plasminogen activators, and fibrin degradation products) in the subdural collection of CSDHs are thought to promote the development and recurrence of CSDH [19,26]. These substances may be contained in the debris of the subdural collection. The most important goal of surgery should be the complete replacement of CSDH liquid with normal saline. In the SBAID group, by using aspiration and irrigation, especially aspiration, subdural collections were almost completely replaced by normal saline, and little debris was left in the subdural space.

The patients were placed in a supine position with the head tilted slightly to the opposite side during the operation. In this way, Wei's point was the highest point of the operative field, and the air was easily evacuated (Figures 2). Therefore, the air volume left in the cavity in the SBAID group was much lower than that in the SBID group (p=0.03).

The data in this paper support the superiority of SBAID compared with SBID with respect to the radiographic resolution of CSDHs. In addition, we show that the SBAID method produces better results in regard to the duration of surgery, the length of hospital stay, and the rate of recurrence at an average follow-up duration of 31 weeks.

Our study does have some limitations that need to be addressed. First, the sample size of this study was small. Second, the study provided only a technical description and, therefore, randomized controlled trials are required to clarify its efficacy. Third, our research was a retrospective study. Therefore, we should perform a large-scale, prospective, randomized controlled trial to verify our strategy.

## Conclusions

Our results indicate that SBAID is a simple, effective, and safe procedure for the treatment of CSDHs. Using Wei's point for the burr hole, we can eliminate the possibility of tension pneumocephalus; using the aspiration method, we can evacuate almost all of the debris of the hematoma. Both of these innovations help significantly reduce the recurrence of CSDH.
